# MEASURING AND IMPLEMENTING PERSON-CENTERED LONG-TERM CARE: GLOBAL PERSPECTIVES

**DOI:** 10.1093/geroni/igad104.0965

**Published:** 2023-12-21

**Authors:** Tonya Roberts, Franziska Zuniga, Michael Lepore

## Abstract

Person-centered care (PCC) is internationally recognized as an essential strategy for achieving high quality care and quality of life for older adults who need long-term care (LTC). Despite international endorsement, our understanding of PCC is largely derived from research and practice in western countries. It is less clear whether PCC practices and benefits are generalizable on a global scale. PCC may vary structurally across healthcare contexts and cultures where values, experiences, and needs of older adults differ. Understanding how to culturally adapt or extend the concept, measurement, and implementation of PCC is needed to better inform care and improve outcomes for a diverse and international population of older adults. The purpose of this symposium is to present theory and research across healthcare settings and from countries across multiple continents to comparatively analyze the cultural implications of PCC concepts and discuss the implications for implementation, measurement, and research. The first presentation will contextualize the concept of PCC from an international perspective, summarizing and comparing what is known about PCC from existing literature. The second presentation will compare barriers and facilitators of PCC implementation during mealtimes in three European countries. The third will highlight variations in PCC definitions from qualitative research conducted with providers in middle- and low-income countries. The fourth presentation will report on the development and implementation of a system of PCC and LTC in Brazil. The final presentation will use secondary data to compare the measurement and LTC needs of family caregivers across the US and China. This is a Common Data Elements for International Research in Residential Long-term Care Interest Group Sponsored Symposium.

